# Synergistic Antifungal Interaction between *Pseudomonas aeruginosa* LV Strain Metabolites and Biogenic Silver Nanoparticles against *Candida auris*

**DOI:** 10.3390/antibiotics12050861

**Published:** 2023-05-06

**Authors:** Laís Fernanda de Almeida Spoladori, Gabriella Maria Andriani, Isabela Madeira de Castro, Helena Tiemi Suzukawa, Ana Carolina Ramos Gimenes, Guilherme Bartolomeu-Gonçalves, Kelly Ishida, Gerson Nakazato, Phileno Pinge-Filho, Rayanne Regina Beltrame Machado, Celso Vataru Nakamura, Galdino Andrade, Eliandro Reis Tavares, Lucy Megumi Yamauchi, Sueli Fumie Yamada-Ogatta

**Affiliations:** 1 Programa de Pós-Graduação em Microbiologia, Universidade Estadual de Londrina, Londrina CEP 86057-970, Brazil; lais.spoladori@gmail.com (L.F.d.A.S.); mgabriella.andriani@gmail.com (G.M.A.); isabela.mcastro@uel.br (I.M.d.C.); helena.tiemi.suzukawa@uel.br (H.T.S.); gnakazato@uel.br (G.N.); pingefilho@uel.br (P.P.-F.); cvnakamura@uem.br (C.V.N.); andradeg@uel.br (G.A.); lionilmy@uel.br (L.M.Y.); 2 Laboratório de Biologia Molecular de Microrganismos, Universidade Estadual de Londrina, Londrina CEP 86057-970, Brazil; ana.carolina.ramos@uel.br (A.C.R.G.); tavares.eliandro@uel.br (E.R.T.); 3 Programa de Pós-Graduação em Fisiopatologia Clínica e Laboratorial, Universidade Estadual de Londrina, Londrina CEP 86038-350, Brazil; guilherme.bartolomeu@uel.br; 4 Laboratório de Quimioterapia Antifúngica, Universidade de São Paulo, São Paulo CEP 05508-000, Brazil; ishidakelly@usp.br; 5 Laboratório de Bacteriologia Básica e Aplicada, Universidade Estadual de Londrina, Londrina CEP 86057-970, Brazil; 6 Laboratório de Imunopatologia Experimental, Universidade Estadual de Londrina, Londrina CEP 86057-970, Brazil; 7 Laboratório de Inovação Tecnológica no Desenvolvimento de Fármacos e Cosméticos, Universidade Estadual de Maringá, Maringá CEP 87020-900, Brazil; raymachado6@hotmail.com; 8 Laboratório de Ecologia Microbiana, Universidade Estadual de Londrina, Londrina CEP 86057-970, Brazil

**Keywords:** antibiofilm activity, antifungal synergism, fluopsin C, green silver nanoparticles, indolin-3-one

## Abstract

*Candida auris* has been found to be a persistent colonizer of human skin and a successful pathogen capable of causing potentially fatal infection, especially in immunocompromised individuals. This fungal species is usually resistant to most antifungal agents and has the ability to form biofilms on different surfaces, representing a significant therapeutic challenge. Herein, the effect of metabolites of *Pseudomonas aeruginosa* LV strain, alone and combined with biologically synthesized silver nanoparticles (bioAgNP), was evaluated in planktonic and sessile (biofilm) cells of *C. auris*. First, the minimal inhibitory and fungicidal concentration values of 3.12 and 6.25 μg/mL, respectively, were determined for F4a, a semi-purified bacterial fraction. Fluopsin C and indolin-3-one seem to be the active components of F4a. Like the semi-purified fraction, they showed a time- and dose-dependent fungicidal activity. F4a and bioAgNP caused severe changes in the morphology and ultrastructure of fungal cells. F4a and indolin-3-one combined with bioAgNP exhibited synergistic fungicidal activity against planktonic cells. F4a, alone or combined with bioAgNP, also caused a significant decrease in the number of viable cells within the biofilms. No cytotoxicity to mammalian cells was detected for bacterial metabolites combined with bioAgNP at synergistic concentrations that presented antifungal activity. These results indicate the potential of F4a combined with bioAgNP as a new strategy for controlling *C. auris* infections.

## 1. Introduction

Fungal infections have a major impact on public health worldwide, especially due to the increase in the number of immunocompromised individuals [1]. Yearly, up to 1.5 million people are estimated to die due to fungal infections [2]. In recent decades, *Candida auris* has emerged as a global threat to human health, causing persistent and difficult-to-treat infections [3,4]. Indeed, isolates of *C. auris* presenting resistance to the main classes of clinically available antifungals (azoles, polyenes, and echinocandins) are increasingly reported worldwide [5,6].

*C. auris* can be found as a colonizer of human skin without disease symptoms. Critically, persistent *C. auris* colonization may increase the risk of subsequent infections, particularly in hospitalized and immunocompromised individuals, who may develop invasive infections with high morbidity and mortality rates [4,7,8]. In addition, asymptomatic carriers may also serve as a source for cross-transmission of *C. auris* among the hospital community and may contaminate surfaces in the hospital environment, facilitating its dissemination. Besides healthcare settings, *C. auris* has been recovered from marine [9] and wastewater environmental samples [10]. In fact, since its first report in Japan [3], this yeast species has quickly disseminated globally [8].

Biofilm formation on different surfaces plays an essential role in *C. auris*’s ability to survive for a long period in humans [7,11] and on abiotic surfaces [12,13,14,15], as well as in the antifungal resistance [12,13,14,16,17]. Notably, this community of cells, firmly adhered (sessile cells) to a surface and encased in a self-produced extracellular polymeric matrix, displays reduced sensitivity to antifungal agents that are active against the planktonic counterpart cells [12,13,14,16,17]. Moreover, biofilm formation is linked with enhanced virulence in the *Galleria mellonela* infection model [12]. Given these alarming characteristics, the World Health Organization has classified *C. auris* as a critical fungal pathogen for which the development of new control strategies is a high priority [18].

Since the discovery of penicillin, a secondary metabolite produced by the fungus *Penicillium notatum*, microorganisms have been one of the most important sources for the discovery of new antibiotics. Indeed, an important number of microbial secondary metabolites and analogs with several pharmacological properties were introduced on the market and are still used in clinical practice. For instance, the macrolide antifungals amphotericin B and nystatin originated from the metabolism of the bacteria *Streptomyces nodosus* and *Streptomyces noursei*, respectively [19]. Due to their remarkable metabolic diversity, species of *Pseudomonas* are ubiquitous in the natural environment. These bacteria produce a wide range of secondary metabolites, some of which have a broad spectrum of antimicrobial activity, which may give them a selective advantage in natural environments [20,21]. Previous studies have shown that secondary metabolites from the culture of *Pseudomonas aeruginosa* LV strain, a bacterium isolated from a foliar lesion of *Citrus sinensis* var Valencia [22], have potent antibacterial activity against phytopathogens [23,24,25,26] and human multidrug-resistant pathogens [27,28,29,30]. Secondary metabolites obtained from *Pseudomonas* spp. strains with inhibitory activity on growth of *Candida albicans* have been previously reported [31,32,33]. However, the antifungal activity of metabolites from *Pseudomonas* spp. on *C. auris* has not been described so far.

Combined therapy approaches to control infections have been another attractive strategy, especially in those caused by multidrug-resistant microorganisms [34,35]. Some advantages may be associated with the use of combined therapy, such as the synergistic effect due to action on multiple targets, a broad spectrum of action, improvement in the bioavailability of one or more compounds, and reduced risk of both the toxicity and the emergence of resistance during treatment [36]. Actually, some antimicrobial combinations are already in clinical use for the treatment of infectious diseases. For instance, intravenous amphotericin B in combination with oral flucytosine is recommended as the standard treatment of cryptococcal meningitis, especially in immunocompromised individuals. This antifungal combination aims to reduce the fungal burden on the host and the risk of treatment failure [37]. From this perspective, the combination of silver nanoparticles (AgNP) with different compounds, including antimicrobials, has been extensively investigated [34,35].

AgNP can be produced by biological, chemical, and physical approaches, involving different methods. Biological synthesis attracts attention for being a simple, eco-friendly, and cost-effective method without toxic chemicals [38,39]. AgNP, biosynthesized using plant [39,40,41] and microbial [39,42,43,44] metabolites, were capable of inhibiting the planktonic and biofilm mode of growth of different *Candida* species, including *C. auris* [40,41].

In the present study, we report for the first time the antifungal activity of secondary metabolites from *P. aeruginosa* LV strain, both alone and combined with biogenic silver nanoparticles (bioAgNP), on planktonic and biofilm cells of *C. auris*. Furthermore, the effect of the bacterial metabolites, both alone or combined with bioAgNP, on the viability of mammalian cells was also evaluated.

## 2. Results and Discussion

### 2.1. F4a and Its Components, Fluopsin C and Indolin-3-One, Inhibit the Growth of Planktonic Cells of Candida auris, Displaying a Dose- and Time-Dependent Fungicidal Effect

Several studies describe the effects of the interaction between *P. aeruginosa* and *Candida* spp., and, in general, this interaction has been characterized as antagonistic to yeast species [21]. This antagonistic interaction may result from competition for the adhesion site on host surfaces, or for nutrient acquisition, or the secretion of molecules that impair the growth of a species [45]. In fact, the inhibitory activity of *P. aeruginosa* metabolites on the growth of planktonic [31,32] and sessile cells [33] of *C. albicans* has been previously reported.

In this study, the antifungal effect of the dichloromethane fraction (F4a) obtained from the cell-free supernatant of *P. aeruginosa* LV strain cultured under copper stress [22,46] was analyzed on planktonic cells of *C. auris* CBS 10913 and *C. auris* CBS 12766 (thereafter, these strains were named CBS 10913 and CBS 12766, respectively). CBS 10913 was sensitive to fluconazole and amphotericin B, whereas CBS 12766 was resistant to both antifungal agents [47]. F4a inhibited the growth of both strains, displaying minimal inhibitory concentration (MIC) and minimal fungicidal concentration (MFC) values of 3.12 and 6.25 μg/mL (Table 1), respectively. F4a is composed of a mixture of four bioactive compounds: the organocopper antibiotic fluopsin C, indolin-3-one, phenazine-1-carboxamide, and phenazine-1-carboxilic acid [26,29]. To identify the antifungal component of F4a, MIC and MFC of each compound were determined. Only fluopsin C and indolin-3-one displayed an inhibitory activity on planktonic cells of *C. auris*. Although it inhibited the planktonic growth of *C. auris*, the MIC and MFC values of indolin-3-one were significantly (*p* < 0.05) higher than those of F4a (Table 1). On the other hand, MIC = 0.78 μg/mL and MFC = 1.56 μg/mL were determined for fluopsin C, and these values were significantly lower (*p* < 0.02) than those of F4a (Table 1), indicating that fluopsin C may be one of the active compounds of F4a toward *C. auris* strains.

Fluopsin C was first isolated and characterized from the culture of *Pseudomonas* MCRL 10107 (named YC 73) [31] and *Pseudomonas fluorescens* KY 403 (named fluopsin C) [48]. Subsequently, fluopsin C was isolated from *Pseudomonas reptilivora* [49] and *Streptomyces* [50], and recently, from *P. aeruginosa* PAO1 [51]. These early studies reported a potent inhibitory activity of fluopsin C against the fungus, *Saccharomyces cerevisiae,* and several Gram-positive and Gram-negative bacteria [31,48,51]. The YC 73 also inhibited the growth of several fungal species, including one *C. albicans* isolate (MIC = 3.12 μg/mL) [31]. Regarding fluopsin C purified from *P. aeruginosa* LV strain, previous studies only reported the inhibition of planktonic cell growth of the multi-drug resistant bacteria, including methicillin-resistant *Staphylococcus aureus*, vancomycin-resistant *Enterococcus faecium*, and *Klebsiella pneumoniae* carbapenemase-producing *K. pneumoniae* (KPC-*K. pneumoniae*) [29,30].

**Figure 1:**
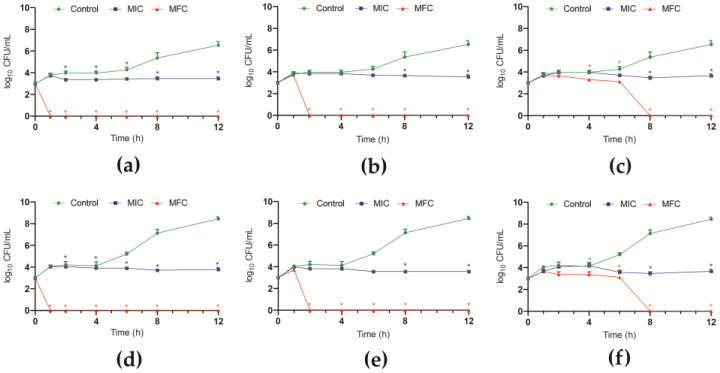
Antifungal activity of secondary metabolites from *Pseudomonas aeruginosa* LV strain in *Candida auris*. Time–kill kinetics of *C. auris* CBS 10913 (**a**–**c**); *C. auris* CBS 12766 (**d**–**f**). The log_10_ CFU/mL values were the mean and the standard deviation representative of three independent experiments. (**a**,**d**) dichloromethane fraction (F4a); (**b**,**e**) fluopsin C; (**c**,**f**) indolin-3-one. * *p* < 0.05.

To assess the time and the nature of the antifungal effect, the growth kinetics of planktonic cells in the presence of F4a, fluopsin C, and indolin-3-one were monitored during 12 h at 37 °C. Overall, at MIC values of *P. aeruginosa* metabolites, an inhibition of growth of *C. auris* strains was observed over time compared to untreated control cells (Figure 1). A dose- and time-dependent fungicidal effect was observed in planktonic cells, and at the MFC, the colony-forming unit (CFU) counts were zero after 1, 2, and 8 h-incubation for F4a (Figure 1a,d), fluopsin C (Figure 1b,e), and indolin-3-one (Figure 1c,f), respectively.

### 2.2. F4a and Indolin-3-One Display Synergistic Interactions with bioAgNP in Planktonic Cells of Candida auris

In the present study, the bioAgNPs were biosynthesized using an aqueous extract from the bark of *Trichilia catigua* Adr. Juss (Meliaceae family). According to the technical report and transmission electronic microscopy (TEM) analysis (Figure S1), these water-soluble nanoparticles displayed spherical morphology around 90–100 nm, which was further confirmed by dynamic light scattering (DLS) analysis (data not shown). In addition, X-ray diffraction (XDR) analysis was performed to determine the crystalline nature of the bioAgNps, and some distinctive peaks at 2*θ*, which are characteristic of AgNPs, were identified (Figure S1).

**Figure 2:**
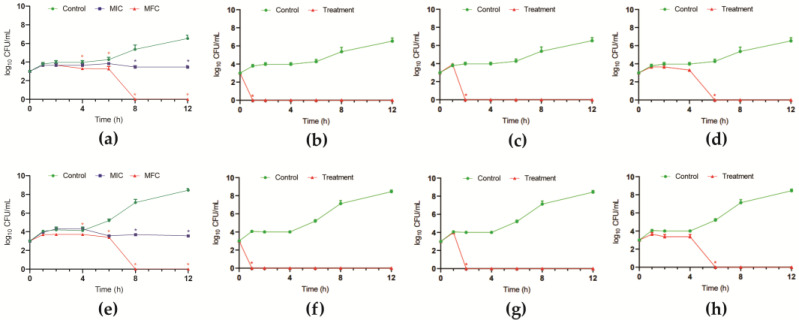
Antifungal interaction of secondary metabolites of *Pseudomonas aeruginosa* LV strain with biogenic silver nanoparticles (bioAgNP) in *Candida auris*. Time–kill kinetics of *C. auris* CBS 10913 (**a**–**d**) and *C. auris* CBS 12766 (**e**–**h**) The log_10_ CFU/mL values were the mean and the standard deviation representative of three independent experiments. (**a**,**e**) bioAgNP alone; (**b**,**f**) dichloromethane fraction (F4a)/bioAgNP; (**c**,**g**) fluopsin C/bioAgNP; (**d**,**h**) indolin-3-one/bioAgNP. * *p* < 0.05.

The MIC and MFC of bioAgNPs were determined and were equal to 6.68 μg/mL and 26.75 μg/mL, respectively, for both *C. auris* strains. The time–kill kinetics showed a time- and dose-dependent fungicidal effect of bioAgNP against both strains. At the MIC value, the nanoparticles inhibited the yeast growth over the analyzed time (12 h), and at the MFC value, the CFU counts were zero after 8 h-incubation (Figure 2a,e).

Next, the interaction of the *P. aeruginosa* metabolites with bioAgNP on *C. auris* planktonic cells was evaluated by the checkerboard assay, and the results are shown in Table 2. Except for fluopsin C, the simultaneous addition of F4a or indolin-3-one with bioAgNP displayed a synergistic antifungal activity according to the fractional inhibitory concentration indices (FICI). Both strains exhibited a 2-fold and a 28-fold decrease in MIC values of F4a and bioAgNP, respectively; and a 4096-fold and a 32-fold decrease in MIC values of indolin-3-one and bioAgNP, respectively. Although the combination of fluopsin C and bioAgNP was not classified as synergistic, there was a 2-fold and a 16-fold reduction in the MIC values of these compounds, respectively (Table 2). Interestingly, the combination of fluopsin C and indolin-3-one also exhibited a synergistic antifungal interaction, causing a 2-fold and a 4096-fold reduction in their MIC values, respectively (Table 2). These results indicate that fluopsin C and indolin-3-one are important for the synergistic fungicidal activity.

The growth kinetics of planktonic cells in the presence of the compound combinations (at MIC) during 12 h at 37 °C revealed a time-dependent fungicidal interaction (Figure 2). Except for the combination between indolin-3-one and bioAgNP, the time–kill kinetic patterns were similar for F4a or fluopsin C combined with bioAgNP. The CFU counts were zero after 1, 2, and 6 h-incubation for F4a (Figure 2b,f), fluopsin C (Figure 2c,g), and indolin-3-one (Figure 2d,h), respectively, at the synergistic combinations with bioAgNP.

A limitation of this study is that the mechanism of antifungal action of the combinations between *P. aeruginosa* LV strain metabolites and bioAgNP was not extensively analyzed. However, microscopy analyses were carried out to gain insights into the mechanism of action of the compound combinations. As there was no difference in MIC and MFC values, as well as in the time–kill kinetics between both yeast strains, CBS 10913 was selected for further analyses, except when specified.

**Figure 3:**
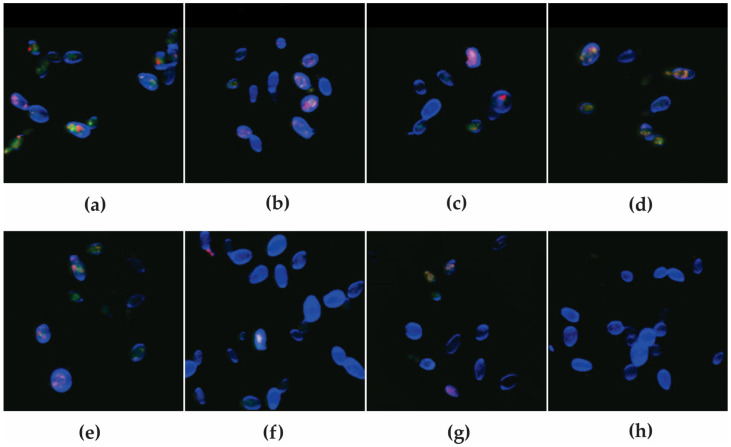
Cell viability analysis of *Candida auris* CBS 10913 after differential labeling with FUN-1™ and Calcofluor White™ MR2. Yeasts were incubated with or without the MICs of the compounds for 24 h. Cells containing red fluorescent intravacuolar structures represent metabolically active yeast and blue fluorescence indicates the cell wall of viable and *non*-viable cells. Cells with diffuse greenish-yellow fluorescence characterize metabolically inactive cells (**a**) untreated planktonic cells; treated cells at MIC with (**b**) dichloromethane fraction (F4a); (**c**) fluopsin C; (**d**) indolin-3-one; (**e**) bioAgNP; (**f**) dichloromethane fraction (F4a)/bioAgNP; (**g**) fluopsin C/bioAgNP; (**h**) indolin-3-one/bioAgNP.

The antifungal effect of *P. aeruginosa* metabolites alone or combined with bioAgNP was visualized after differential labeling of CBS 10913, using the fluorescent probes FUN-1™ and Calcofluor™ White M2R (Figure 3). The images corroborated the fungistatic activity of all metabolites alone. Figure 3a shows the untreated planktonic cells with typical yeast-like morphology, intact cell wall and plasma membrane, and reddish vacuolar structures, indicating intact membrane and metabolic activity [53]. Similarly, at the MIC values of F4a (Figure 3b), fluopsin C (Figure 3c), and indolin-3-one (Figure 3d), no morphological changes were observed, and we see the presence of red-orange intravacuolar structures, corroborating the fungistatic nature of the compounds in these concentrations. Conversely, F4a (Figure 3f), fluopsin C (Figure 3g), and indolin-3-one (Figure 3h) combined with bioAgNP at the synergistic concentrations displayed a diffuse green fluorescence or absence of reddish fluorescence, indicating the presence of metabolically inactive cells with damaged membranes. As previously reported, F4a is a semi-purified fraction consisting of 25% of fluopsin C and indolin-3-one, and according to our results, the interaction of both metabolites seems to be crucial for the observed fungicidal activity. Moreover, owing to lower production cost of F4a resulting from fewer required purification steps when compared to isolated compounds [26,46], F4a combination with bioAgNP was selected for further analyses.

**Figure 4:**
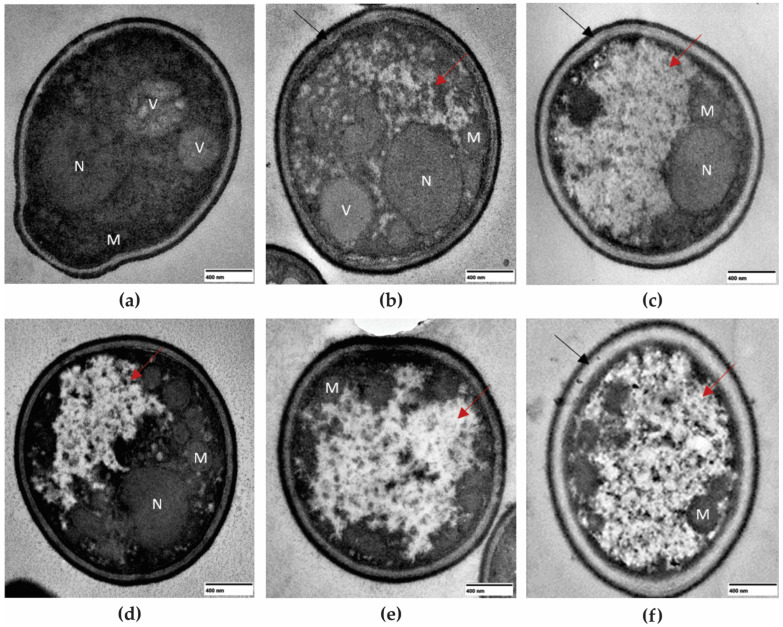
Transmission electron microscopy images of *Candida auris* CBS 10913 after 24 h of treatment with dichloromethane fraction (F4a) and biogenic silver nanoparticles (bioAgNP) alone or combined. (**a**) Untreated control cells; treatment with (**b**) 3.12 μg/mL F4a; (**c**) 6.25 μg/mL F4a; (**d**) 6.68 μg/mL bioAgNP; (**e**) 26.75 μg/mL bioAgNP; (**f**) 1.56/0.05 μg/mL F4a/bioAgNP. Nucleus (n); vacuole (v); mitochondria (m); cytoplasmic disorganization (red arrow); and cell membrane detachment (black arrow).

The TEM images showed ultrastructural changes in planktonic cells of CBS 10913 after treatment with MIC and MFC of F4a, bioAgNP, and the combination of both compounds (Figure 4). Untreated cells (Figure 4a) displayed intact cell wall and plasma membrane, cytoplasm organization, and the presence of intact mitochondria and vacuoles. After 24 h of treatment with MIC values of F4a (Figure 4b) and bioAgNP (Figure 4d), a slight decrease in electron density and disorganization of cytoplasm were observed, and these alterations were more intensified after the treatment of yeast cells with MFC values of both compounds (Figure 4c,e). Intense disorganization of the cytoplasm and impairment of organelles, in addition to cell membrane detachment, were observed in yeast cells treated with the combination of both compounds at the synergistic concentration (Figure 4f).

Studies on the mechanisms of action of F4a and fluopsin C, as well as AgNPs alone, have been described in the literature. Navarro et al. [54] reported that the plasma membrane of Gram-positive and Gram-negative bacteria had been identified as the primary target of F4a and fluopsin C. The treatment of bacterial cells with these compounds affected the cell wall compaction, cell membrane permeabilization, and divisional septum and decreased the cytoplasmic electron density [29,30].

Regarding bioAgNPs, the antifungal effect strongly depends on size, shape, and coating agents. Given this, most of the knowledge about the mechanisms of action of silver nanoparticles has been obtained using chemically synthesized silver nanoparticles (AgNP). These nanoparticles attach to and accumulate on the fungal surface by electrostatic interaction and are actively transported inside the cell [55]. Once inside the cell, AgNP can lead to increased intracellular reactive oxygen species (ROS) [56], and alterations in multiple targets such as cell wall integrity, membrane permeability and fluidity, mitochondria metabolism, fatty acid composition, protein denaturation, and DNA damage [39,57,58]. Few studies have evaluated the antifungal effect of biogenic AgNP against *C. auris*. Mare et al. [40] described the antifungal activity of bioAgNP synthesized by using *Picea abies* L (*Pa*-bioAgNP, average size of 75.91 nm) bark aqueous extract (enriched with polyphenol compounds) on *C. auris* CBS 10913, and MIC_50_ and MIC_100_ values were equal to 80.0 and 160.0 μg/mL, respectively. The same authors observed no synergistic antifungal interaction with fluconazole on *C. auris*. The study of Malik et al. [41] analyzed the antifungal effect of bioAgNP synthesized by using an aqueous extract (enriched with polyphenol compounds) of the plant *Cynara cardunculus* (*Cc*-bioAgNP, average size of 26.89 nm) on *C. auris* MRL6057, a clinical strain resistant to amphotericin B. The MIC and MFC values of *Cc*-bioAgNP were equal to 50.0 and 100.0 μg/mL, respectively; these nanoparticles induced cell cycle arrest, mitochondrial membrane depolarization, and DNA fragmentation, resulting in cell death by apoptosis. On the other hand, bioAgNP synthesized by using an aqueous extract from the bark of *Fagus sylvatica* L. (average size of 32.0 nm) did not inhibit the growth of planktonic cells of *C. auris* CBS 10913 [59].

Compared with our results, bioAgNPs synthesized using the aqueous extract of *T. catigua* seem to have greater potency against *C. auris*, judging by the MIC and MFC values. New studies aimed at elucidating the fungicidal mechanism of the interaction between F4a and bioAgNP should be performed to support the potential of this combination to control *C. auris* infection.

### 2.3. Candida auris Strains form Dense Biofilms on Abiotic Surfaces and bioAgNP Enhances the Antibiofilm Activity of F4a, Displaying Synergistic Interactions

The search for new antifungal compounds with antibiofilm activity is one of the main strategies to combat resistant microorganisms and infections associated with biofilm formation. In fact, microbial biofilms are predominantly found in natural environments and contribute to the pathogenesis of numerous microorganisms in different hosts [60]. Crucially, biofilms-related infections are difficult to treat and seem to be associated with high mortality rates in patients with candidemia [61].

**Figure 5:**
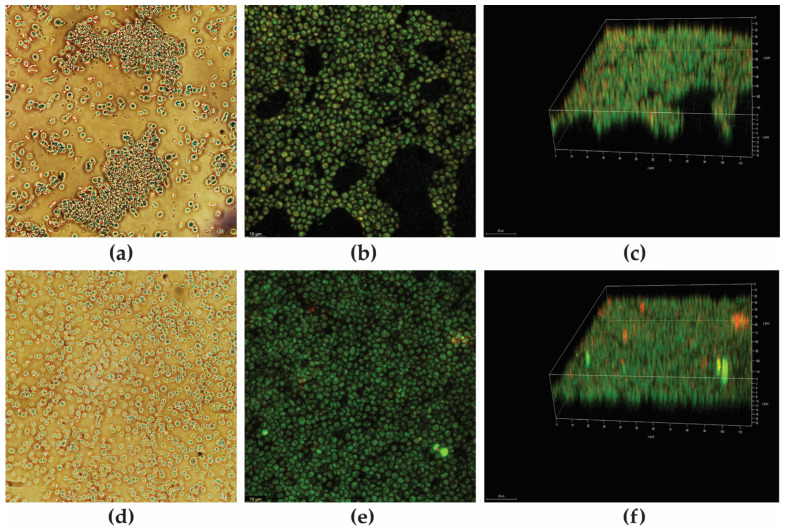
Microscopy images of planktonic cells and biofilms of *Candida auris*. Light microscopy images of (**a**) aggregate-forming *C. auris* CBS 10913 and (**d**) non-aggregate-forming *C. auris* CBS 12766 in phosphate-saline buffer suspensions. 1000× magnification. Confocal laser scanning microscopy images of (**b**,**c**) *C. auris* CBS 10913 and (**e**,**f**) *C. auris* CBS 12766 biofilms formed on glass surface after 24 h at 37 °C and labelled with FUN-1™. (**b**,**e**) Panoramic view of biofilm. Bar: 10 μm. (**c**,**f**) Three-dimensional biofilm reconstitution. Bar: 20 μm.

Previous studies have shown that biofilm formation can vary according to *C. auris* strains [12,14,15]. Given this, we evaluated the biofilm-forming ability of *C. auris* strains on a polystyrene surface. First, we evaluated the aggregation capacity of planktonic cells of both yeast strains. CBS 10913 formed large aggregates of cells mixed with single cells (Figure 5a), whereas CBS 12766 formed homogeneous suspensions after vigorous vortex mixing (Figure 5d) and were classified as aggregative and non-aggregative (single-celled), respectively, according to the criteria by Borman et al. [62]. Although the relationship between single-celled and aggregative phenotypes and biofilm formation is not fully understood, studies have shown that both morphotypes were capable of forming biofilm on abiotic surfaces [12,14,62,63]; non-aggregative strains displayed greater virulence in *Galleria mellonella* killing assays [12,62]; the aggregative phenotype was related to the up-regulation of biofilm-associated genes [63] and the greater ability to survive in abiotic surfaces for a prolonged period [13]; colonizing strains were predominantly aggregative with higher biofilm-producing ability compared to strains isolated from candidemia [14].

In the present study, there was no significant difference (*p* > 0.05) in the amount of 24 h-biofilm biomass formed on polystyrene surface by both *C. auris* strains. The biomass of 24-h biofilms was measured after crystal violet staining, and the optical density at 570 nm (OD_570nm_) ± standard deviation values were 1.192 ± 0.228 and 0.911 ± 0.195 for CBS 10913 and CBS 12766, respectively. Moreover, substantial metabolic activity of sessile cells was observed in 24-h biofilms and remained high up to 72 h, as assessed by the 3-(4,5-dimethylthiazol-2-yl)-2,5-diphenyltetrazolium bromide (MTT) reduction assay (Figure S2). CLSM analysis showed that the biofilms of both strains formed on glass surface consisted of a dense network of yeast cells connected to each other and firmly adhered to the glass surface (Figure 5b,c,e,f). However, CBS 10913 appears to form a thinner biofilm in this surface (18-μm-thick biofilms, Figure 5c) compared to CBS 12766 (20-μm-thick biofilms Figure 5f).

**Figure 6:**
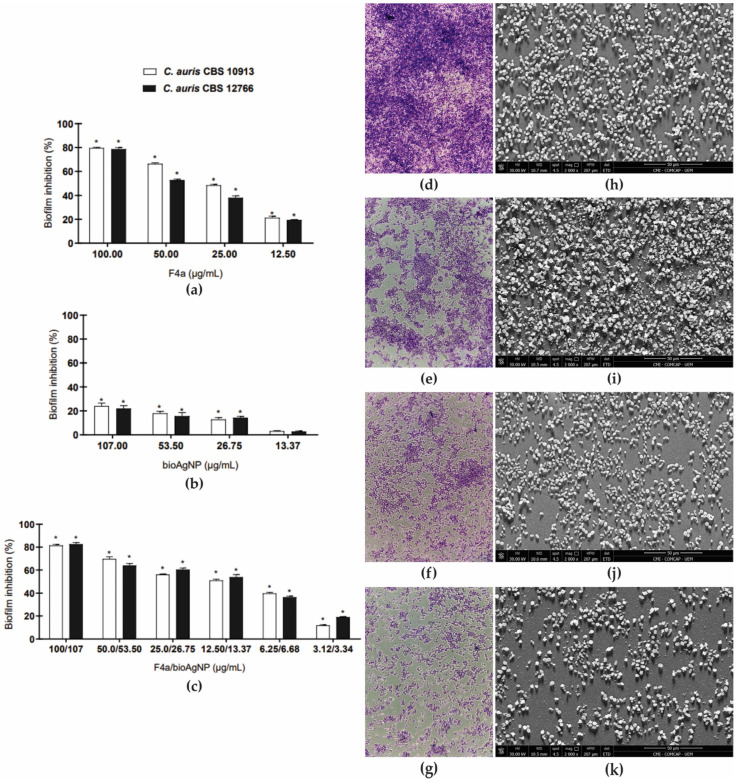
Antifungal activity of dichloromethane fraction (F4a) and biogenic silver nanoparticles (bioAgNP) alone or combined on 24-h biofilm of *Candida auris*. (**a**–**c**) The effect of the compounds on sessile cells was evaluated by colony forming units counting after 24 h of incubation at 37 °C. The values were converted in percentage, considering the untreated biofilms as controls. Values are mean ± standard deviation of two experiments in quintuplicate and were analyzed by one-way ANOVA. Asterisks indicate a significant percentage inhibition of treated-biofilms (*p* < 0.05) compared to untreated ones. (**a**) F4a; (**b**) bioAgNP; (**c**) F4a/bioAgNP. (**d**–**g**) Light microscopy (1000× magnification) and (**h**–**k**) scanning electron microscopy (bar: 50 μm) images of *C. auris* CBS 10913 biofilms on polystyrene during 24 h of incubation at 37 °C. (**d**,**h**) Untreated control; (**e**,**i**) 100.0 μg/mL F4a; (**f**,**j**) 107.0 μg/mL bioAgNP; (**g**,**k**) 12.40/13.21 μg/mL F4a/bioAgNP.

Next, the effects of F4a alone and combined with bioAgNP were evaluated on 24-h biofilms of *C. auris* strains. A dose-dependent inhibition of the viability of sessile cells (evaluated by CFU counts) of both yeast strains was observed for F4a (Figure 6a). After 24 h of treatment, the minimal inhibitory concentrations required to reduce 50.0% of CFU counts (SMIC_50_) were 32.94 and 47.02 μg/mL for CBS 10913 and CBS 12766, respectively (Table 3 and Figure 6a). The SMIC_80_ for both strains was 100.0 μg/mL. The antibiofilm effect of *P. aeruginosa* LV metabolites has been little explored. Kerbauy et al. [28] reported a significant reduction in the biomass of 24-h biofilms of the bacterium KPC-*K. pneumoniae*, after treatment with the planktonic cells MIC value (62.5 μg/mL) of F3d (a dichloromethane fraction enriched with phenazine-1-carboxyamide and fluopsin C, and other minor components [23]). In addition, an antibiofilm activity was also observed in 24-h biofilms of *Staphylococcus aureus*, including those exhibiting methicillin-resistance, with F4a SMIC_50_ ranging from 12.5 to 25.0 μg/mL [30].

Interestingly, bioAgNP alone presented low inhibitory activity on sessile cells, even at the highest concentration tested (Table 3 and Figure 6b). The bioAgNP (107.0 μg/mL) caused less than 25.0% reduction in CFU counts of 24 h-biofilms of both *C. auris* strains after 24 h of treatment. In contrast to our results, the study of Vazquez and Munoz et al. [64] reported SMIC values ranging from 0.5 to 4.9 μg/mL of chemically AgNP coated with polyvinylpyrrolidone (PVP, average size of 6.18 ± 5 nm) for *C. auris* from different clades. Similarly, AgNP synthesized by microwave irradiation-assisted heating reaction (average size of 1.0 to 3.0 nm) inhibited the biofilm formation (SMIC_50_ = 60.0 ng/mL) and 24 h-biofilms (SMIC_50_ = 480.0 ng/mL) of *C. auris* [65]. The PVP-coated AgNP also synthesized by microwave irradiation (average size of 15.0 to 20.0 nm) inhibited the biofilm formation, and the SMIC_50_ ranged from 0.7 to 3.2 μg/mL [66]. Thus, these discrepant results could be explained by the differences in the size and coating agents among the nanoparticles.

Different combinations of F4a and bioAgNP concentrations caused a dose-dependent inhibitory activity on 24 h-biofilms of both *C. auris* strains (Figure 6c). The treatment of 24-h biofilms with F4a combined with bioAgNP decreased the SMIC_50_ values of all compounds for both strains (Table 3). The interaction of F4a with bioAgNP on biofilms was classified as synergistic, with calculated FICI values of 0.49 and 0.29 for CBS 10913 and CBS 12766 strains, respectively (Table 3).

Untreated and treated 24 h-biofilms of CBS 10913 formed on polystyrene surfaces were analyzed by light microscopy (Figure 6d-g) and scanning electron microscopy (SEM, Figure 6h-k). The untreated control biofilm consisted of a dense network of yeast cells with typical rounded morphology attached to the abiotic surface (Figure 6d,h). In contrast, treatment with F4a SMIC_80_ (Figure 6e,i) and bioAgNP (107.0 μg/mL Figure 6f,j) and the combination of both at the synergistic concentration (Figure 6g,k) showed a significant decrease in the number of cells within the biofilms and intense yeast cell damage. Taken together, these results reinforce the antifungal potential of *P. aeruginosa* LV strain metabolites in combination with bioAgNP in *C. auris*.

### 2.4. Bacterial Metabolites Combined with bioAgNP Does Not Cause Toxicity to LLC-MK2 Cells at the Fractional Inhibitory Concentration on Planktonic Cells

Previous studies have shown that the cytotoxic concentration of F4a and fluopsin C able to inhibit the viability of 50% (CC_50_) of the LLC-MK2 cells (kidney epithelial cells from *Macaca mulatta*) was equal to 3.44 and 2.0 μg/mL, respectively, which was close to the MIC values determined for planktonic cells of *C. auris* in the present study. Moreover, at concentrations greater than 6.25 μg/mL of bacterial metabolites, no viable LLC-MK2 cells were detected [29,30]. In the present study, the CC_50_ of indolin-3-one for LLC-MK2 cells was 71.69 μg/mL, and at the MIC value (100.0 μg/mL), approximately 42.0% of the cells were viable (data not shown). Regarding AgNP, toxicity to mammalian cells seems to depend on the release of silver ions; the toxic effects can be influenced by different factors, such as synthesis method, capping agent, size, morphology, and concentration [67]. Here, the incubation of different concentrations of bioAgNP did not interfere with the spectrometric analysis of the MTT-reduction assay. Thus, the CC_50_ of bioAgNP for LLC-MK2 cells was 6.33 μg/mL, also close to the MIC value for planktonic cells of *C. auris*. At concentrations equal to or higher than 13.3 μg/mL, bioAgNP inhibited the metabolic activity of about 95.0% of the tested cells (data not shown).

**Figure 7:**
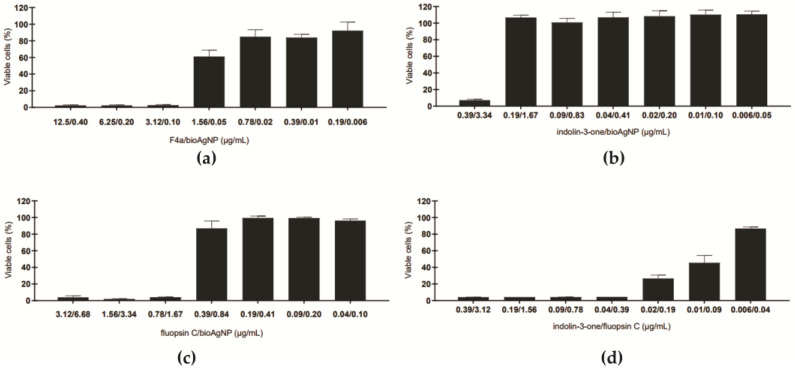
Effect of secondary metabolites from *Pseudomonas aeruginosa* LV strain and biogenic silver nanoparticles (bioAgNP) on metabolic activity of LLC-MK2 cells after 24 h-incubation. Values are mean ± standard deviation of two experiments in duplicate. (**a**) dichloromethane fraction (F4a)/bioAgNP; (**b**) indolin-3-one/bioAgNP; (**c**); fluopsin C/bioAgNP; (**d**) indolin-3-one/fluopsin C.

To determine whether the combination of bacterial metabolites and bioAgNP could cause toxicity to mammalian cells, the viability of LLC-MK2 cells was analyzed after incubation with several compound concentration combinations. The results are shown in Figure 7. All combinations at the minimal inhibitory concentration values were less toxic for LLC-MK2 cells when compared to the compounds alone. At the synergistic concentrations of F4a (1.56 μg/mL) and bioAgNP (0.05 μg/mL) (Figure 7a) or indolin-3-one (0.04 μg/mL) and bioAgNP (0.41 μg/mL) (Figure 7b), 61.0% and 100.0% of viable cells were observed, respectively. For the combination of fluopsin C (0.39 μg/mL) and bioAgNP (0.84 μg/mL), metabolic activity was observed in about 86.0% of the cells (Figure 7c). On the other hand, the synergistic concentration of indolin-3-one (0.04 μg/mL) and fluopsin C (0.39 μg/mL) decreased the metabolic activity of about 96.0% of the cells (Figure 7d). These results reinforce the potential of fluopsin C and indolin-3-one as a starting point for the development of new antifungal agents for *C. auris*. Further studies are necessary to achieve the following objectives: (a) reduce toxicity to mammalian cells, which may involve modifying the chemical structure of the active components of F4a, exploring the use of drug delivery systems and investigating bioAgNP synthesized by different biological reducing agents; (b) evaluate the toxicity of compound combinations in vivo to corroborate the in vitro results.

## 3. Materials and Methods

### 3.1. Microorganisms and Growth Conditions

*P. aeruginosa* LV strain was isolated from an old canker lesion on leaves of *Citrus sinensis* cv. Valence [22]. The bacterium was stored in nutrient broth containing 30% glycerol (*v*/*v*) at −80 °C in the Microbial Culture Collection of the Laboratory of Microbial Ecology of the Universidade Estadual de Londrina, Paraná, Brazil. For the experiment, the bacterium was cultured in nutrient agar supplemented with 0.01% CuCl_2_.2H_2_O, pH 6.8 at 28 °C for 48 h.

*C. auris* CBS 10913 [3] and *C. auris* 12766 [4] were stored in Sabouraud dextrose (SD) broth containing 30% glycerol (*v*/*v*) at −80 °C. For the experiments, the yeast strains were cultured in SD agar at 37 °C for 24 h. Afterward, three to five colonies were cultured in SD broth, under the same conditions to prepare a standard yeast suspension. Thus, yeast cells were harvested by centrifugation (10,000× *g* for 1 min) and resuspended in 0.85% NaCl solution (saline) to a turbidity equivalent to 0.5 McFarland standard (approximately 1.0–2.0 × 10^6^ CFU/mL) using the DensiCHEK™ PLUS colorimeter (bioMérieux). The standard yeast suspension was further diluted in a culture medium to achieve the inoculum used in each assay.

### 3.2. Purification of Metabolites from Pseudomonas aeruginosa LV Strain

The secondary metabolites of *P. aeruginosa* LV strain were obtained through the patented methodology [22] modified by Bedoya et al. [46]. Briefly, log-phase *P. aeruginosa* cells were cultured in nutrient broth supplemented with 0.01% CuCl_2_ 2H_2_O pH 6.8 at 28 °C for 10 days. Then, the cells were harvested by centrifugation (4500× *g*, 20 min), and the supernatant was used for subsequent purification procedures. To obtain the fraction 4a (F4a), the supernatant was extracted twice with dichloromethane, followed by purification by flash chromatography with silica gel 60 (0.063–0.200 mm, Merck, São Paulo, Brazil) using a mobile phase of dichloromethane:ethyl acetate (1:1, *v*/*v*). To obtain fluopsin C, indolin-3-one, phenazine-1-carboxyamide, and phenazine-1-carboxylic acid, F4a was further purified by flash chromatography with silica gel 60 (0.04–0.062 mm, Marcherey-Nagel, Rio Grande do Sul, Brazil), using petroleum ether:dichloromethane:ethyl ether (65:25:10) as the mobile phase. Next, a semi-preparative Agilent 1260 Infinity high-performance liquid chromatography (HPLC) was performed using a gradient of acetonitrile:water as mobile phase. Stock solutions of 4.0 mg/mL of all products were prepared in 100% dimethylsulfoxide (DMSO, Merck, São Paulo, Brazil). DMSO did not exceed 1.25% in all assays.

### 3.3. Biologically Synthesized Silver Nanoparticles

Biogenic silver nanoparticles (bioAgNP) were acquired from GRAL Bioativos^®^ LDTA (Brazil), which were obtained after the reduction of AgNO_3_ by *Trichilia catigua* Adr. Juss bark aqueous extract. The biosynthesis and characterization (by TEM, XDR and DLS) of the bioAgNPs were carried out according to the patented methodology [BR1020210163755, http://www.inpi.gov.br (accessed on 4 March 2023)] [68].

### 3.4. Antifungal Activity against Planktonic Cells

#### 3.4.1. Minimal Inhibitory (MIC) and Fungicidal (MFC) Concentrations Determination

The MIC of *P. aeruginosa* metabolites and bioAgNP was determined by the broth microdilution technique according to the Clinical and Laboratory Standards Institute [69]. Tests were carried out in Roswell Park Memorial Institute 1640 (Gibco Co., São Paulo, Brazil), plus 0.165 M morpholine propane sulfonic acid (RPMI-MOPS) with a final yeast inoculum equivalent to 0.5 × 10^3^–2.5 × 10^3^ CFU/mL. The compound concentrations ranged from 0.19 to 100.0 μg/mL for bacterial metabolites, 0.021 μg/mL to 107.0 μg/mL for bioAgNP. *Candida parapsilosis* ATCC 22019, fluconazole (Gemini Indústria de Insumos Farmacêuticos Ltd., São Paulo, Brazil) and amphoterecin B (Merck, São Paulo, Brazil) were included in each experiment as quality control. For each U-bottom 96-well plate (Techno Plastic Products, Switzerland), two wells were used as growth (medium *plus* 1.25% DMSO *plus* yeast cells) and sterility (medium *plus* 1.25% DMSO) controls. MIC was defined as the lowest concentration capable of inhibiting visual growth after 24 h of incubation at 37 °C compared to growth control. The susceptibility breakpoints for the antifungals were those recommended by the Centers for Disease Control and Prevention [6] of the United States of America. To determine the MFC of bacterial metabolites and bioAgNP, an aliquot of 10 μL from the wells without visible growth was inoculated onto the SD agar. The MFC was determined as the lowest concentration capable of reducing the CFU counts to zero after 24 h at 37 °C. The experiments were carried out in duplicate on three different occasions.

#### 3.4.2. Checkerboard Microdilution Assay

The antifungal effect of bacterial secondary metabolites combined with bioAgNP on planktonic cells was evaluated using the checkerboard broth microdilution assay [70]. Two-fold serial dilutions of F4a (0.001 to 3.12 μg/mL) or fluopsin C (0.0003 to 0.78 μg/mL) or indolin-3-one (0.04 to 100.0 μg/mL) and bioAgNP (0.003 to 107.0 μg/mL), respectively, were added across the rows and columns of the U-bottom 96-well microtiter plates. Thereafter, yeast cells (1.0 × 10^3^ CFU/mL) were added, and the plates were incubated at 37 °C for 24 h. The Fractional Inhibitory Concentration (FIC) of each compound was determined by the ratio of the MIC obtained when the compounds were tested in combination and the MIC of the compounds tested individually. The FIC Index (FICI) was calculated from the sum of the FIC_secondary metabolites_ and FIC_bioAgNP_, and the values were interpreted as follows: synergistic (FICI ≤ 0.5), no interaction (0.5 < FICI < 4.0), or antagonistic (FICI > 4.0) [52].

#### 3.4.3. Time–Kill Kinetics

The rate of *C. auris* killing in the presence of bacterial secondary metabolites alone or combined with bioAgNP was analyzed by time–kill assay [71]. The yeast cells (1.0 × 10^3^ CFU/mL) were added to the wells of U-bottom 96-well microtiter plates containing RPMI-MOPS supplemented with bacterial metabolites and bioAgNP alone (at MIC and MFC values) and combined (at MIC values). The plates were incubated at 37 °C, and, at specified time points (0, 1, 2, 4, 6, 8 and 12 h), the CFU counts were determined. Thus, a 10 μL-aliquot was removed from each well and serially diluted (1:10) in saline, and 10 μL of each dilution was inoculated onto SD agar. The CFU counts were carried out after incubation at 37 °C for 24 h. Averaged data were plotted as log_10_ CFU/mL versus time (h). The fungicidal effect of the compounds was defined as a 99.9% (3 log_10_) reduction in CFU/mL of the starting inoculum [72].

#### 3.4.4. Yeast Cell Viability

Viability of planktonic yeast cells was evaluated using the LIVE/DEAD™ Yeast Viability Kit (Molecular Probes, Invitrogen, São Paulo, Brazil) according to the manufacturer’s recommendations. *C. auris* (1.0 × 10^7^ CFU/mL) strains were treated with the bacterial secondary metabolites and bioAgNP alone (at MIC values) or combined (at MIC values) at 37 °C for 24 h. Afterward, the yeast cells were incubated with FUN-1™ (10.0 μM) and Calcofluor White™ MR2 (25.0 μM) at room temperature for 30 min and examined by confocal laser scanning microscope (TCS SP8 Leica Microsystems) using DAPI (excitation/emission wavelengths of 365/408–480 nm), Alexa Fluor™ 555 (excitation/emission wavelengths of 550/568–630 nm), and FITC (excitation/emission wavelengths of 500/550–555 nm) filters. The images were processed by the LAS X Life Science Office software (Leica Microsystems).

#### 3.4.5. Transmission Electron Microscopy

Planktonic cells (1.0 × 10^7^ CFU/mL) were treated with MIC and MFC values of F4a and bioAgNP alone or combined at the synergistic concentration for 24 h at 37 °C. Untreated and treated yeast cells were fixed with 2.5% glutaraldehyde in 0.1 M sodium cacodylate buffer, pH 7.2, at room temperature for 2 h. Then, the post-fixation was carried out in 0.2 M cacodylate buffer, pH 7.2, containing 2.0% osmium tetroxide, 1.6% potassium ferrocyanide, and 10 mM CaCl_2_ for 30 min at room temperature. The cells were dehydrated in acetone and embedded in Epon resin. Ultrathin sections were stained with uranyl acetate and lead citrate and examined with a FEI Tecnai T12 transmission electron microscope.

### 3.5. Antifungal Activity against Sessile (Biofilm) Cells

#### 3.5.1. Aggregation Behavior Analysis

The aggregation capacity of *C. auris* was evaluated as described by Borman et al. [62]. Briefly, *C. auris* was cultured on SD agar at 37 °C for 24 h, and then the colonies were resuspended in 0.15 M phosphate-buffered saline (PBS), pH 7.2. The cell suspension was vigorously mixed by vortexing for 1 min. A 50 μL-aliquot was transferred on a glass slide, visualized under the light microscope (Olympus CKX53 microscope) at 1000× magnification.

#### 3.5.2. Biofilm Formation

The capacity of *C. auris* strains to form biofilm on an abiotic surface was evaluated on flat-bottomed 96-well polystyrene plates (Techno Plastic Products, Switzerland), as described by Bizerra et al. [73]. The biofilms were formed in SD broth statically at 37 °C for 12, 24, 48, and 72 h, with an inoculum of 1.0 × 10^7^ CFU/mL. After each incubation period, the viability of sessile cells was evaluated using the MTT (Merck, São Paulo, Brazil) reduction assay according to the manufacturer’s recommendations. The assays were carried out in quintuplicate and performed on two separate occasions.

The 24-h biofilms of *C. auris* were analyzed using CLSM. The biofilms were formed on glass coverslips (9 mm in diameter) immersed in wells of 24-well cell culture plates (Techno Plastic Products, Trasadingen, Switzerland) containing 1.0 mL of SD broth, as aforementioned. The biofilms were gently washed once with PBS, pH 7.2. Subsequently, the biofilms were treated with 4 μL of PBS containing FUN-1™ (10.0 μM, Molecular Probes, Invitrogen, São Paulo, Brazil) and incubated for 2 h at room temperature. The biofilms were examined using a laser scanning microscope with the Alexa Fluor™ 555 and FITC filters, as described above.

#### 3.5.3. Antifungal Activity

The biofilms of *C. auris* were formed on flat-bottomed 96-well polystyrene plates as described above, and after 24 h-incubation at 37 °C, non-adherent cells were removed by washing once with PBS. A 200-μL aliquot of RPMI-MOPS containing different concentrations of F4a (12.5 to 100.0 μg/mL) or bioAgNP (13.37 to 107.0 μg/mL) was added to determine the sessile minimal inhibitory concentration (SMIC) of each compound.

The antifungal interactions between F4a and bioAgNP on biofilms were evaluated according to Brilhante et al. [74]. Briefly, the biofilms were formed on flat-bottomed 96-well polystyrene plates for 24 h, as described above. Each bacterial metabolite and bioAgNP were, previously, two-fold serially diluted; then, aliquots (50 μL) of each concentration were transferred to biofilms. The compounds were tested at the aforementioned concentration ranges. After 24 h of incubation at 37 °C, the biofilms were washed once with PBS, pH 7.2.

The viability of sessile cells was determined by the CFU counts [75]. Thus, untreated- and treated biofilms were removed by scraping with a sterile scalpel. Sessile cells were resuspended in PBS, sonicated for 5 min, and then vortexed for 30 s. The cell suspensions were ten-fold serially diluted, 100 μL-aliquots were inoculated on SD agar, and the plates were incubated at 37 °C for 24 h. CFU counts were carried out to estimate the total number of viable cells. The values were converted into percentages and used to determine the lowest concentration capable of inhibiting 50% of the sessile cells (SMIC_50_). The results of the combination were interpreted using the FICI as described above. The assays were carried out in triplicate and performed on two separate occasions.

#### 3.5.4. Microscopy Analyses

Morphological alterations provoked by the bacterial metabolites and bioAgNP alone or combined on *C. auris* biofilms were analyzed by light microscopy and scanning electron microscopy (SEM). For light microscopy, the biofilms and treatments were carried out as described previously. The biofilm biomass was fixed with 99.9% methanol (15 min), dried at room temperature, and stained with 0.02% (*w*/*v*) crystal violet for 20 min. After washing with PBS, the stained biofilm was observed under an Olympus CKX53 microscope. For SEM analyses, the strips of polystyrene (0.5 cm^2^) were immersed in wells of 24-well cell culture plates containing 1.0 mL of SD broth, and then the biofilm was formed as described above. The 24-h biofilms were treated with compounds alone (SMIC_80_) or combined at the synergistic concentration at 37 °C for 24 h. The biofilms were fixed with 2.5% (*v*/*v*) glutaraldehyde in 0.1 M sodium cacodylate buffer pH 7.2 at room temperature, dehydrated with serial ethanol washes (30, 50, 70, 80, 90, 95, and 100%), critical point dried in CO_2_, coated with gold, and observed in a FEI Quanta 250 scanning electron microscope.

### 3.6. Effect of Pseudomonas aeruginosa Secondary Metabolites and bioAgNP on Mammalian Cells

The cytotoxicity of bacterial secondary metabolites and bioAgNP alone or combined was evaluated on kidney epithelial cells from *Macaca mulatta* (LLC-MK2 cells (Merck, São Paulo, Brazil)). Cells (1.0 × 10^4^ cells/well) were cultured in RPMI supplemented with 10% fetal bovine serum (Invitrogen, São Paulo, Brazil), 2 mM L-glutamine, 100 IU/mL penicillin, 100 μg/mL streptomycin, and 1% tylosin in flat-bottomed 96-well microtiter plates in 5% CO_2_ at 37 °C for 48 h. Then, the medium was carefully aspirated-off and fresh media containing (8-, 4-, 2-, 1-, 1/2-, 1/4- and 1/8-fold of MIC value) bacterial metabolites and bioAgNP, alone or combined, were added. The cells were incubated for a further 24 h under the same conditions. Cell viability was analyzed by the MTT reduction assay according to the manufacturer’s recommendation. The concentration of the compound needed to inhibit the viability of 50% of the cells calculated by regression analysis corresponds to the 50% (CC_50_) cytotoxic concentrations. To evaluate the interference of bioAgNP on spectrometric analysis [76], several concentrations of nanoparticles (0.005 to 107.0 μg/mL) were directly incubated with MTT, and the assay was carried out according to the manufacturer’s recommendations.

### 3.7. Statistical Analyses

The GraphPad PRISM software version 8.0 (GraphPad Software, San Diego, CA, USA) was used for statistical analyses. Time–kill kinetics and toxicity to LLC-MK2 cells were analyzed using two-way ANOVA followed by Tukey’s multiple comparisons test. The biofilm results were evaluated by one-way ANOVA. For all assays, *p* < 0.05 was considered significant.

## 4. Conclusions

The present study reports for the first time the antifungal activity of a semi-purified fraction (F4a) containing secondary metabolites of *P. aeruginosa* LV strain produced under copper stress, both alone and combined with biologically synthesized silver nanoparticles (bioAgNP) using *T. catigua* bark aqueous extract on planktonic and sessile (biofilm) cells of *C. auris*. Fluopsin C and indolin-3-one seem to be the active components of F4a. Moreover, F4a combined with bioAgNP exhibited a potent fungicidal and synergistic interaction against planktonic cells, causing intense cellular damage at non-toxic concentrations to mammalian cells. The combination between F4a and bioAgNP also inhibited the 24 h-biofilms of *C. auris*, decreasing the number of viable cells within these fungal communities. This study has some limitations that may reduce the generalizability of its results: (a) the number of fungal strains tested is limited and do not represent all clades of *C. auris*; (b) all tests were carried out under in vitro conditions, and in vivo studies are required to assess the efficacy of antifungal activity and mammalian toxicity to support the in vitro results; (c) the mechanism of action of the compound combination has not yet been fully elucidated. Despite these limitations, the results indicate that the combination of F4a and bioAgNP may be a promising prototype for developing new strategies to control *C. auris* infections.

## Figures and Tables

**Table 1:** Antifungal activity of secondary metabolites of *Pseudomonas aeruginosa* LV strain on planktonic cells of *Candida auris* CBS 10913 and *Candida auris* CBS 12766.

Compounds	^1^ MIC (μg/mL)	^2^ MFC (μg/mL)
F4a	3.12	6.25
Fluopsin C	0.78	1.56
Indolin-3-one	100	200

^1^ MIC: Minimal inhibitory concentration. ^2^ MFC: Minimal fungicidal concentration. F4a, dichloromethane fraction.

**Table 2:** Antifungal interaction between secondary metabolites of *Pseudomonas aeruginosa* LV strain and biogenic silver nanoparticles on planktonic cells of *Candida auris* CBS 10913 and *Candida auris* CBS 12766.

Combination	^1^ MIC_S_ (μg/mL)	^2^ MIC_C_ (μg/mL)	FICI	Interaction
F4a/bioAgNP	3.12/6.68	1.56/0.05	0.50	Synergism
fluopsin C/bioAgNP	0.78/6.68	0.39/0.84	0.62	Indifferent
indolin-3-one/bioAgNP	100/6.68	0.04/0.41	0.06	Synergism
indolin-3-one/fluopsin C	100/0.78	0.04/0.39	0.50	Synergism

^1^ MICs: Minimal inhibitory concentration singly. ^2^ MIC: Minimal inhibitory concentration in combination. FICI: Fractional inhibitory concentration index. F4a, dichloromethane fraction; bioAgNP, biogenic silver nanoparticles. Reference values: synergism, FICI ≤ 0.5; antagonism, FICI ≥ 4 and indifferent, FICI > 0.5 to 4 [52].

**Table 3:** Antifungal interaction of dichloromethane fraction of *Pseudomonas aeruginosa* LV strain and biogenic silver nanoparticles on 24 h-biofilms of *Candida auris* CBS 10913 and *Candida auris* CBS 12766.

Strain	^1^ F4a (μg/mL)	^1^ bioAgNP (μg/mL)	^1^ F4a/bioAgNP (μg/mL)	FICI	Interaction
CBS 10913	32.94	>107.0	12.40/13.21	0.49	Synergism
CBS 12766	47.02	>107.0	9.26/10.21	0.29	Synergism

^1^ SMIC_50_: Minimal inhibitory concentration capable of inhibiting the metabolic activity of 50% of sessile cells; FICI: Fractional inhibitory concentration index; F4a, dichloromethane fraction; bioAgNP, biogenic silver nanoparticles. Reference values: synergism, FICI ≤ 0.5; antagonism, FICI ≥ 4 and indifferent, FICI > 0.5 to 4 [52].

## Data Availability

Not applicable.

## Supplementary Materials

The following supporting information can be downloaded at: https://www.mdpi.com/article/10.3390/antibiotics12050861/s1, Figure S1: Biological silver nanoparticles synthesized by using the aqueous bark extract from *Trichilia catigua* characterization. Figure S2: Kinetics of biofilm formation by *Candida auris* on polystyrene surface monitored by measuring the metabolic activity of sessile cells using the MTT reduction assay.
