# Supplementary material for: Synergistic Antifungal Interaction between Pseudomonas aeruginosa LV Strain Metabolites and Biogenic Silver Nanoparticles against Candida auris
Source: Antibiotics (Basel). 2023 May 6;12(5):861. doi: 10.3390/antibiotics12050861 (PMC10215102; doi:10.3390/antibiotics12050861)
Supplement: Supplementary file 1 [file antibiotics-12-00861-s001.zip › antibiotics-2346753-supplementary.pdf]

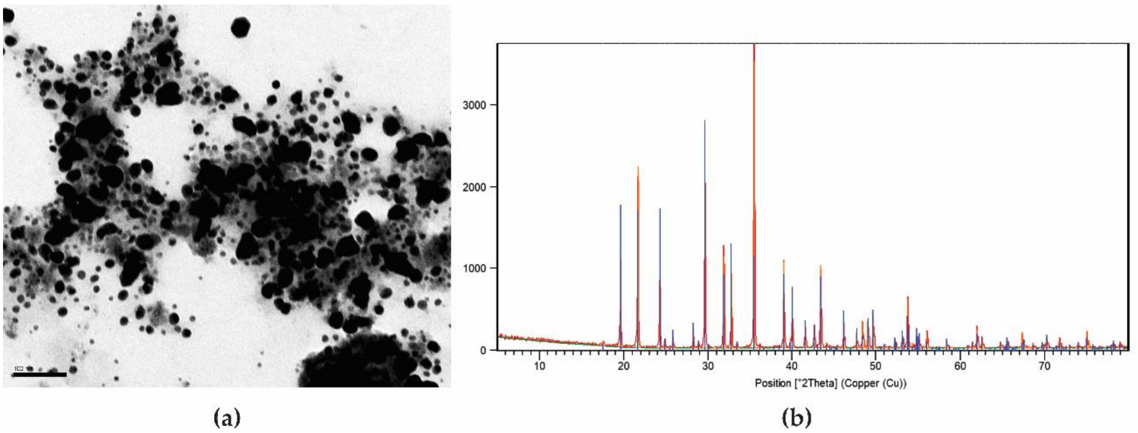

Figure S1: Biological silver nanoparticles synthesized by using the aqueous bark extract from *Trichilia catigua* characterization by (**a**) transmission eletronic microscopy (TEM) and (**b**) X-ray diffraction (XDR) analysis. (**a**) TEM image shows silver nanoparticles with spherical morphology around 90-100 nm. (**b**) XDR image shows the characteristic peaks of silver nanoparticles.

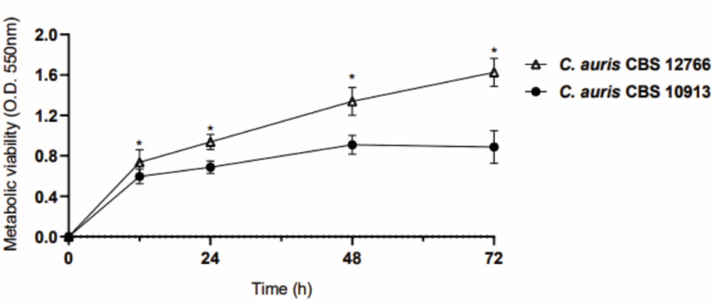

Figure S2: Kinetics of biofilm formation by *Candida auris* on polystyrene surface monitored by measuring the metabolic activity of sessile cells using the MTT reduction (OD_{550nm}) assay. The values represent the mean ± standard deviation of three independent experiments. Asterisks indicate significant difference (p < 0.05) between metabolic activity of the strains.
